# Explaining interindividual differences in toddlers' collaboration with unfamiliar peers: individual, dyadic, and social factors

**DOI:** 10.3389/fpsyg.2015.00493

**Published:** 2015-05-01

**Authors:** Nils Schuhmacher, Joscha Kärtner

**Affiliations:** Department of Developmental Psychology, University of MünsterMünster, Germany

**Keywords:** toddler, peer collaboration, problem-solving task, commitment to joint activities, coordination, mothers' expectations, temperament, mastery motivation

## Abstract

During their third year of life, toddlers become increasingly skillful at coordinating their actions with peer partners and they form joint commitments in collaborative situations. However, little effort has been made to explain interindividual differences in collaboration among toddlers. Therefore, we examined the relative influence of distinct individual, dyadic, and social factors on toddlers' collaborative activities (i.e., level of coordination and preference for joint activity) in joint problem-solving situations with unfamiliar peer partners (*n* = 23 dyads aged *M* = 35.7 months). We analyzed the dyadic nonindependent data with mixed models. Results indicated that mothers' expectations regarding their children's social behaviors significantly predicted toddlers' level of coordination. Furthermore, the models revealed that toddlers' positive mutual experiences with the unfamiliar partner assessed during an initial free play period (Phase 1) and their level of coordination in an obligatory collaboration task (Phase 2) promoted toddlers' preference for joint activity in a subsequent optional collaboration task (Phase 3). In contrast, children's mastery motivation and shyness conflicted with their collaborative efforts. We discuss the role of parents' socialization goals in toddlers' development toward becoming active collaborators and discuss possible mechanisms underlying the differences in toddlers' commitment to joint activities, namely social preferences and the trust in reliable cooperation partners.

## Introduction

The last decades have seen an ongoing interest in a social behavior, cooperation, of which humans, among vertebrate species, provide particularly outstanding examples. In recent years, human cooperation has also become a fundamental topic in developmental and comparative psychology: Researchers are increasingly investigating our children's and our closest ancestors' (i.e., great apes) cooperative skills and motivations. This focus has stressed human unity (e.g., compared to great apes) and amply demonstrated the complexity of human cooperation, but generally downplayed the extant individual differences. So the major concern of the present study is to rectify this lacuna by answering the question: How can we further explain interindividual differences in early human cooperation, namely in peer collaboration among toddlers?

To conceptually describe the phenomenon of cooperation, we shall classify it according to the scheme proposed by Melis and Semmann (2010). Following these authors, cooperative behaviors (or cooperation) can be subsumed as social behaviors among two (or more) agents that either bring an immediate benefit to another conspecific (the recipient) or to both agents as an outcome of their joint coordinated actions (by the recipient and the actor). The former type of behaviors is typically called altruistic cooperation (e.g., helping, sharing, or comforting); the latter, mutualistic cooperation. Based on these definitions, collaboration is an instance of mutualistic cooperation or simply mutualism (see Tomasello et al., 2012; Warneken and Melis, 2012).^1^ More specifically, we shall refer to an elementary form of collaboration within the scope of this article, that is, to dyadic (i.e., small-scale) cooperation with immediate and synchronous benefits for both interaction partners.

Much of the previous and concurrent (developmental) research on cooperation has used comparative psychology to investigate the phylogenetic and ontogenetic roots of human altruistic and mutualistic behaviors (Tomasello et al., 2005, 2012; Warneken and Tomasello, 2009a,b; Jaeggi et al., 2010; Silk and House, 2011; Liebal and Haun, 2012; Warneken and Melis, 2012). This central line of research postulates that humans are naturally prepared and motivated to cooperate from early on in their lives (e.g., Tomasello et al., 2005; Warneken and Tomasello, 2009a,b). In particular, at the beginning of the second year of life, toddlers already possess the essential cognitive and motivational prerequisites for collaborative activities. For example, 2- to 3-year-old children clearly prefer to act together with a peer partner to master a joint problem-solving task (i.e., reaching food) even if they have the alternative opportunity to act individually in order to attain a similar goal. In contrast, chimpanzees do not prefer to collaborate in these situations (Rekers et al., 2011). Various other findings support the assumption that young human children are already skillful collaborators, whereas our nearest relatives (chimpanzees and bonobos) collaborate to a comparably limited degree (e.g., Warneken et al., 2006; see also Warneken and Melis, 2012, for a review). Based on this empirical fundament of predominantly experimental comparative studies, some researchers conclude that mutualistic cooperation is perhaps a uniquely human capacity and, furthermore, the cradle of norms, morality, and human culture (e.g., Tomasello, 2009; Tomasello et al., 2012; Rakoczy and Schmidt, 2013).

Most research so far analyzed the development of human cooperation by contrasting either children at different ages or children to their nearest primate relatives to learn more about the age-typical developmental progression, most importantly the age of emergence. Corresponding findings are usually interpreted along the lines of great-divide theories (e.g., 2-year-old humans do cooperate, chimps do not) in order to make general conclusions about the phylogenetic roots of the investigated competencies. By doing so, the current discourse lost track of another core aspect of development, namely interindividual differences in early cooperation and the individual and contextual factors that play an important role in shaping toddlers' cooperative behavior. As can be observed in toddler's everyday encounters, there is a substantial variance in their social interactions with others. Stated differently, even though young children have already acquired the necessary cognitive and motivational competencies to collaborate with others, this does not determine how they actually behave socially or guarantee that cooperation will be a success. In particular, different factors might interfere with or support children's intrinsic motivation to cooperate and their actual performance in cooperative situations. These potential influences range from individual factors (e.g., temperament), over dyadic factors (i.e., those describing the characteristics of a dyad), to generally social factors (e.g., the role of parents in fostering toddlers' cooperative behavior). However, up to now, little effort has been invested in explaining these interindividual differences in toddlers' collaborative activities. Thus, this study aims to identify the relative contribution of individual, dyadic, and social factors to children's collaboration. Before elaborating each of these factors, we shall specify the two key aspects of dyadic collaboration, namely coordination and commitment to joint goals.

Children's competencies in coordinating their actions in collaborative social play or problem-solving situations are subsumed under the heading of coordination. During their second and third years of life, toddlers increasingly come to coordinate their actions with others and start to collaborate successfully. Whereas 18-month-olds are already quite proficient in coordinating their activities with an adult partner in different social play and cooperative problem-solving situations (Ross and Lollis, 1987; Warneken et al., 2006), it is not before 2–3 years of age that they start coordinating their actions and can successfully master joint problem-solving tasks with a peer partner (Brownell and Carriger, 1990; Ashley and Tomasello, 1998; Brownell et al., 2006). This ontogenetic gap (or peer gap) might be explained by differences in sociocognitive or motivational demands in adult–child compared to peer collaboration, for example, because adults show higher levels of scaffolding and greater competencies in collaborative situations (Brownell, 2011). Moreover, previous studies on adult-child collaboration were rather structured than naturalistic, that is, the adult orchestrated the interaction with the child following a constrained script (see also Warneken and Melis, 2012, p. 410).

Based on philosophical accounts on shared collaborative activities (e.g., Bratman, 1992; Tuomela, 2007). Tomasello (2009) emphasizes a specific aspect of collaboration: Social interaction partners act together in a “we” mode rather than in an “I” mode, that is, they have formed a *shared intentionality* which includes a joint goal and a common plan regarding how to work together in order to achieve that joint goal (see also Tomasello et al., 2005). In particular, humans are specifically motivated to establish shared intentional states and consequently to work jointly toward shared goals with others (Tomasello et al., 2005; Gräfenhain et al., 2009). Moreover, Tomasello et al. (2012) denote that “[…] once they have formed a shared goal, humans are committed to it.” (p. 677; see also Bratman, 1992). This commitment toward a joint goal implies that the goal is stable over time and that there is an obligation toward the partner to fulfill this goal by acting together. Remarkably, toddlers already indicate these motivational prerequisites for joint collaborative activities, that is, they are motivated to share psychological states and act jointly just for the sake of acting together with another person. In addition, they potentially feel an obligation to persist in this mutual activity with the partner, that is, they are committed to the joint activity. This was demonstrated in various recent studies on adult-child and peer interactions (see also Warneken and Melis, 2012, for a review): As one early indicator for toddlers' understanding of joint commitments, Warneken et al. (2006) found that 18- and 24-month-olds showed reengagement attempts directed toward an adult partner, that is, verbal and nonverbal imperatives directed toward the experimenter when the recalcitrant partner suddenly stopped the joint activity. Some previous studies on adult-child interaction proclaim that there needs to be an explicit agreement of both interactions partner toward the joint activity, in order to establish a joint commitment (e.g., adult enforces explicit commitment of the child: “Let's play together!”; child responds: “Okay!”; see Gräfenhain et al., 2009). However, a recent study on joint commitment in a peer collaboration context demonstrated that the commitment to a joint activity does not necessarily need to be established explicitly, but can also be established “on the fly,” that is, by acting together (Hamann et al., 2012; see also Tomasello et al., 2012): In the corresponding study, 3.5- (but not 2.5-) year-olds continued to help an “unlucky” peer partner to retrieve a desired reward in a collaborative problem-solving task, even when they had been “lucky” themselves and had already been able to retrieve their own reward before. In particular, they were more willing to provide help in a collaborative compared to a baseline condition and there was no explicit agreement of the peer partners on acting together. Finally, an essential behavioral criterion of joint commitment (that was used in a previous study by Gräfenhain et al., 2009) is the following: If two persons are committed to a joint activity, then one partner should not suddenly stop the joint activity and go on with an individual action instead (as he/she maybe would like to do). Stated differently, there is an obligation of acting together in a “we” mode rather than goal attainment in an “I” mode. In accordance with these assumptions the rationale for assessing toddlers' commitment toward a joint goal in the present study is to investigate their preference for and persistence in joint collaborative activities, that is, to see if and for how long toddlers prefer to act together even when they alternatively have the opportunity to act individually to obtain an identical goal.

The main aim of the present study is to identify the relative contribution of individual, dyadic, and social factors in explaining interindividual differences in children's collaboration, that is, (a) children's competencies in coordinating their actions in collaborative problem-solving situations (i.e., their coordination) and (b) their motivation to participate and persist in collaborative activities (i.e., their preference for joint activity). In the following, we derive four central factors from the current literature, namely mothers' expectations regarding toddlers' cooperation, recent experiences with an unfamiliar peer collaboration partner, toddlers' temperament, and mastery motivation.

One promising candidate for explaining interindividual differences in children's collaboration is the role of specific social factors, for example, parents' expectations toward their offspring's cooperative behaviors. Current positions stress the phylogenetic roots of cooperation and claim that the early development of altruistic and mutualistic activities during infancy and toddlerhood is universal and builds on a biological predisposition (e.g., Warneken and Tomasello, 2009a; Callaghan et al., 2011). Consequently, previous studies paid little attention to individual differences, for example, based on social influences until at least 3 years of age. The assumption is, that socio-cultural influences might set in (and build on the biological predispositions) with children's transition to group-mindedness during preschool age (i.e., between 3 and 6 years of age). Ontogenetically this is the time when children get increasingly concerned about social norms and internalize culture-specific conventions (Tomasello et al., 2012; Rakoczy and Schmidt, 2013). However, first hints from cross-cultural research support the assumption that social factors might already impact on the early development of children's collaboration: Eckermann and Whitehead (1999) found cross-cultural differences in coordinated behaviors in peer interactions (i.e., nonritualized reciprocal games) between a Seltaman (Papua New Guinea people) and a US sample of 8- to 23-month-old children. Rogoff (2003) discusses several findings and concludes that many cultures vary particularly in the ways parents and communities encourage their children to participate in collaborative (group) activities from relatively early on in life. Moreover, in a cross-cultural study by Callaghan et al. (2011), 2-year-olds differed in their motivation to collaborate with an adult partner across cultures: Indian and Peruvian toddlers showed significantly more reengagement attempts in different social play and joint problem solving tasks than Canadian toddlers. Furthermore, in interviews, Indian and Peruvian mothers emphasized the importance of collaboration more than Canadian mothers. Based on these findings it is reasonable to assume that variance in children's collaboration can be found not only between but also within cultures. Moreover, this variance might be accounted for by different socialization experiences (e.g., parents' expectations regarding their child's social behaviors) in 2- to 3-year-old toddlers. However, corresponding empirical evidence is still missing, and there is a lack of studies that systematically analyze the relations between social factors and toddlers' cooperative behavior.

Toddlers' collaborative efforts (i.e., their coordination and preference for joint activities) might also depend on specific characteristics of the interaction partners within a dyad (i.e., level of familiarity or previously shared positive experiences). This is most obvious in interactions among friends, that is, partners with a high level of familiarity: Preschoolers show more positive social behaviors and better conflict resolution in friend compared to nonfriend dyads (e.g., Doyle et al., 1980; Hartup et al., 1988; see also Rubin et al., 2005, for a review). Friends or familiar dyads usually have a history of iterated positive interactions (long-term experiences) and thus presumably form expectations of reciprocity that are likely to promote their motivation to cooperate (e.g., Olson and Spelke, 2008; Moore, 2009). Within dyads of unfamiliar peers, spontaneous positive interactions with the unfamiliar partner—potentially indicating mutual liking—might be associated with collaboration. Young children show a high degree of behavioral variance in interactions among unfamiliar peers: They have positive interactions with some unfamiliar peer partners but not with others; that is, they interact more with peers they spontaneously seem to like (Gottman and Graziano, 1983). Moreover, it is argued that liking among unfamiliar interaction partners potentially increases the probability of collaboration between these partners in short-term interactions (Sigmund, 2009). Cohen and Haun (2013) found that 5- to 10-year-old children were more likely to cooperate with a relatively unfamiliar (puppet) partner if they liked the puppet and previously marked it as a “friend” (compared to a nonfriend puppet). Second, it is reasonable to assume—in an exploratory manner—that previous success during joint-problem solving should increase toddlers' preference for joint activities in a corresponding problem-solving task thereafter. Initial success might be particularly important in unfamiliar dyads, as both partners have no shared history of joint collaborative activities. Hence, the initial performance (and likely associated experiences) might quicken the subsequent preference to act jointly with the partner. That is, if each partner within a dyad discovers that they perform together quite well (or poorly) in a given situation (i.e., collaborate successful in a coordinated manner), this will potentially influence their subsequent motivation to collaborate. However, most previous studies assessed peer collaboration among familiar peer dyads and/or neglected the role of contemporary interaction experiences within the dyads. Up to now, no single study has looked at the joint effect of these two dyadic factors, namely, previous positive experiences with an unfamiliar peer and previous performance in coordinating a joint activity, in order to explain interindividual variation in toddlers' preference for joint activities with an unfamiliar peer partner.

In addition there are important individual factors that are likely associated with toddlers' collaborative behavior. While previous studies particularly focused on socio-cognitive factors (i.e., self-other differentiation and intention understanding; see Brownell and Carriger, 1990; Brownell et al., 2006; Hunnius et al., 2009), other individual factors, that is, toddlers' temperamental dispositions and mastery motivation, were hardly considered in earlier attempts to explain the variability in young children's collaborative activities. Important temperamental correlates might be toddlers' sociablility, shyness (as main indicators of surgency-extraversion) and inhibitory control (as a central indicator of effortful-control; Rothbart et al., 2001; Putnam et al., 2006). Less withdrawn and more out-going children might be socially more competent and thus potentially more collaborative. For example, there are significant associations of shyness and sociability with toddlers' altruistic cooperative behaviors: Less shy and more sociable 3- to 5-year-olds were more willing to instrumentally assist an unfamiliar adult (Stanhope et al., 1987). In addition, inhibitory control seems to be an important correlate of toddlers' general social competences (e.g., Vaughan Van Hecke et al., 2007) and the inhibition of non-functional (e.g., goal irrelevant) behaviors might be particularly important in collaborative contexts (Rothbart and Hwang, 2005). However, up-to-date corresponding empirical evidence on associations with toddlers' mutualistic cooperation is absent.

Finally, this study explores the relation between toddlers' mastery motivation and collaborative behavior. Mastery motivation is particularly interesting, because it is conceptualized as an “intrinsic psychological force that stimulates an individual to attempt independently, in a focused and persistent manner, to solve a problem or master a skill or task which is at least moderately challenging […]” (Morgan et al., 1990, p. 319; see also Morgan et al., 2009)^2^. In addition, mastery motivation is a precursor of later achievement motivation and it increases significantly during toddlerhood, namely between 12 and 36 months of life (Carter et al., 2003). More specifically, traditional mastery motivation research in young children focused on toddlers' persistence and pleasure in autonomously mastering object-oriented tasks (e.g., mastering toys and mechanical devices, effect production, means-ends problem solving, etc.) rather than social mastery, that is, the motivation to generate, maintain, and influence the course of social interactions (e.g., Harter, 1975; Busch-Rossnagel, 1997; Wang and Barrett, 2012). Based on this conceptualization of mastery motivation it seems at hand that an intrinsic motivation to independently master a specific problem solving task (i.e., individual goal attainment) likely conflicts with a motivation for working together along shared goals (i.e., joint intentionality).

To summarize, the main goal of this study is to identify the relative contribution of social, dyadic, and individual factors in explaining interindividual variation in two central aspects of 3-year-olds' collaboration with an unfamiliar peer, namely coordination and commitment to joint activities by preferring and persisting in collaborative (instead of individual) actions. We hypothesized that: (H.1) Mothers' expectations regarding their toddlers' cooperative behaviors will be positively associated with toddlers' collaboration; (H.2) toddlers' previous positive interactions with a peer partner (assessed during an initial free play episode) and experiences of success in a joint problem-solving task will be positively associated with their subsequent motivation to collaborate; and (H.3) toddlers' temperamental factors (i.e., shyness, fear, low inhibitory control) and mastery motivation will be negatively associated with their collaborative behavior.

## Method

### Sample and design

We tested 23 dyads (*N* = 46 children; 15 girls) of older 2-year-olds (mean age: *M* = 35.7 months; *SD* = 1.7; range: 33.2–39.4 months; 9 mixed-sex dyads, 14 same-sex dyads). Two additional dyads had to be dropped from the analysis due to technical problems. All testing took place in a quiet laboratory room. Children were paired with an unfamiliar peer partner. Parents sat in the observation room at a table and were instructed to remain passive during the whole test session by reading magazines or filling out questionnaires. Children were recruited via local registry offices in a medium-sized German city. In terms of SES, participants came from various socio-economic backgrounds, but the sample was representative for German urban educated middle-class families regarding net household income (median = 2500–3000€ per month on a 5-point scale; range: less than 1000€—more than 3000€) and education (24% of the mothers had a general secondary school qualification, 36% had university entrance qualifications, and 40% had a university degree). Parents received 20€ for their child's participation. They were informed about the goals of the present study and gave their written consent on the study participation. The study was conducted in accordance with all applicable laws and rules regarding psychological research in Germany. As part of a funding project of the DFG (German Research Founding Association) a corresponding Committee approved this study. We used a quasi-experimental design and observed toddlers' behaviors during free play (Phase 1) and in two (semi-structured) cooperation situations (Phase 2: obligatory collaboration, Phase 3: optional collaboration). The whole session lasted approximately 40 min and was videotaped.

### Materials

#### Apparatus for obligatory collaboration (Phase 2)

**Figure 1:**
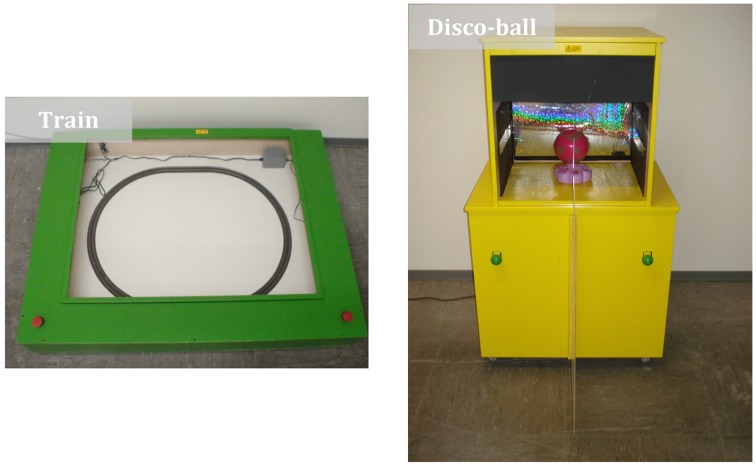
**The two different types of apparatus (*collaboration boxes*) used for the problem-solving task in Phase 2 (left: *train box*, right: *disco-ball box*)**.

In Phase 2, we used two structurally equivalent problem-solving tasks with parallel roles (*collaboration box*): Thirteen dyads played with a (green) train apparatus (*collaborative train box*) and 10 dyads played with a (yellow) disco-ball apparatus (*collaborative disco-ball box*; see also Figure 1). Dyads were allocated randomly to the two different collaboration boxes. In each case, children had to collaborate in order to activate the corresponding toy inside the box. The *collaborative train box* consisted of a green wooden box (1.2 m × 0.8 m × 0.2 m) with a transparent cover plate and a toy train inside. The box lay flat on the floor. Two red buttons were mounted on top of the box. The two buttons had to be pushed simultaneously to activate the train inside the box. Buttons were too far apart to allow individual activation. The *collaborative disco-ball box* consisted of a yellow wooden box with a transparent show window (1.2 m × 0.6 m × 1.5 m) that stood upright in the room. Children had to pull two green handles simultaneously to activate the disco ball behind the show window. Once activated, the ball started to spin while playing music and emitting colorful light. The handles were slightly below the children's shoulder height and could be moved freely back and forth (i.e., in and out of the box). To activate the toy, both handles needed to be pulled fully. Furthermore, there was a transparent Plexiglas divider mounted vertically on the front face of the box, separating the two green handles. This prevented the children from reaching both handles simultaneously; that is, it prevented an individual activation of the ball inside the box.

#### Apparatus for optional collaboration (Phase 3)

**Figure 2:**
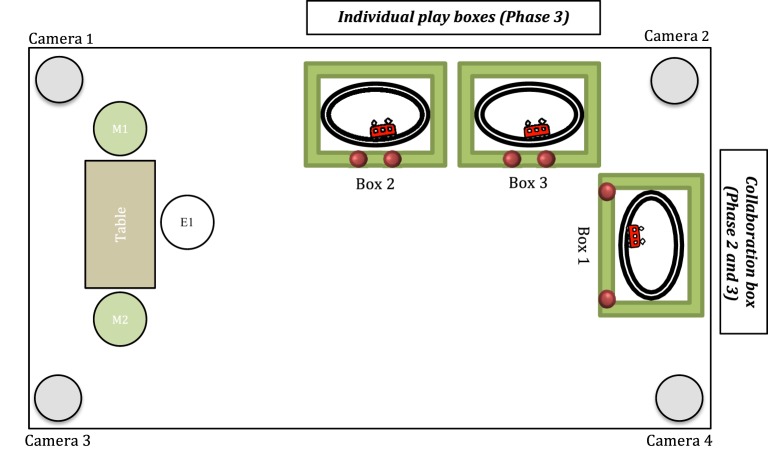
**The general testing room setup**. M1 and M2, mothers; E1, experimenter. All boxes were initially covered with blankets (i.e., at the beginning of the session and during free play in Phase 1). Box 1 (*collaboration box*) was uncovered in Phase 2. Buttons were too far apart to allow individual activation during this phase. Boxes 2 and 3 (*individual play boxes*) were uncovered in Phase 3. The additional boxes were identical to the first box, except for the buttons that were mounted closer together allowing children to activate the toy individually. The setup was identical for the disco-ball boxes.

In Phase 3, two additional boxes were uncovered. These two new boxes were identical to the first box; the only difference being that children could activate the toys inside the boxes individually (*individual play boxes*). The *individual train boxes* had red buttons mounted closer together allowing children to activate the train individually. The *individual disco-ball boxes* had no Plexiglas divider preventing children from triggering the disco ball inside individually. The general setup of Phase 2 and Phase 3 is also illustrated in Figure 2.

### Procedure

The experimenter (E1) welcomed the parents and their children to the lab room. While a second experimenter (E2) instructed the mothers, E1 built up a rapport with the two children. Mothers were instructed to remain passive during the whole test session and encouraged to read a magazine to demonstrate their nonavailability. After these instructions, E2 left the room to operate the cameras in an adjacent video control room. Meanwhile E1 presented a standard set of toys (i.e., building bricks, dolls, balls, cars) to the children and initiated a joint play with them (e.g., building a tower with the bricks). After this warm-up period of approximately 5 min, the experimenter retired discretely and invited the children to carry on playing with the toys together (“I've got to go and sit at the table and read something. You can carry on playing together!”). He finally joined the mothers at the table, sat down, and started reading a magazine (see also Figure 2).

#### Phase 1: Free play

We observed children's behavior during a free play period (Phase 1) for approximately 10 min. Children could play with a standard set of toys (i.e., several building bricks, 2 balls, 2 dolls, 4 cars) during this phase. Mothers and the experimenter remained passive during the whole free play episode. After free play, E1 tidied up the room and all the toys were taken away.

#### Phase 2: Obligatory collaboration

Phase 2 started immediately after the free play. The obligatory collaboration task was modeled similar to Brownell et al. (2006) or Kärtner et al. (2014). E1 initially invited toddlers to join him and uncovered the collaboration apparatus (Box 1 in Figure 2). E1 demonstrated the different parts of the box (i.e., buttons/handles and train/disco-ball inside) to the children. However, he did *not* show them how to activate the toy inside at this point. In the following, E1 increasingly encouraged children to work together according to the following schedule: In *Stage 0* (free problem solving; max. 1 min.), E1 told the children that they could play with the apparatus (“You can play with this!”), went back to the table, and sat down. Children had the opportunity to find out how to activate the toy inside on their own. If they failed to activate the toy, in *Stage 1* (indirect verbal instructions; 1 min), E1 demonstrated how to activate the toy inside the box three times in succession commenting his actions (e.g., “If you pull both handles at the same time, the disco ball will begin to play”). He finally encouraged the children to play with the box: “You can play with this box together. You can try it jointly.” If this did not succeed, during *Stage 2* (direct verbal instructions; 1 min), E1 demonstrated once more how to activate the apparatus. E1 then used more direct verbal instructions to engage children: “Both of you have to push the button/pull the handle to activate the train/ball. Can you help each other?” Finally, if necessary, in *Stage 3* (explicit direction; 1–2 min), E1 explicitly directed children to act jointly and assisted the children by positioning them in the right places to perform their corresponding roles to activate the toy inside: “You (Child A) have to push/pull here, and you (Child B) have to push/pull here!.” Stage 3 was administered infrequently, occurring in less than one-third of dyads. Phase 2 was either stopped 1 min after the occurrence of the toddlers' first successful collaboration *or* after a maximum of 5 min of unsuccessful play.

#### Phase 3: Optional collaboration

At the beginning of Phase 3, the experimenter uncovered the two new boxes (i.e., the individual play boxes) and invited the children to join him (E1: “Shall I show you something? Here are two more boxes!”). He demonstrated the boxes to the children, for example: “Look! These two boxes are similar to that box. But the buttons are much closer together.” E1 activated the toy inside each new box just once. Finally, E1 invited the children to play with the boxes (“You can play with the boxes”) and went back to the table. Children's free activities were videotaped for the following 5 min. The mothers and the experimenter remained passive during the whole phase.

### Coding and measures

The video recordings of toddlers' activities during free play (Phase 1), obligatory collaboration (Phase 2), and optional collaboration (Phase 3) were coded both generally and specifically. The specific coding of the central collaboration variables (i.e., coordination in Phase 2, preference for joint activity in Phase 3) and the different factors are explained in more detail in the following paragraphs. To calculate interrater reliabilities, two raters independently coded 15% of the sample.

#### Level of coordination (Phase 2)

Similar to previous studies, we coded each toddler's level of *coordination* on a 4-point scale for Phase 2 (Warneken et al., 2006; Warneken and Tomasello, 2007; Kärtner et al., 2014). This coding was based on the toddler's (un)coordinated actions in the final stage; that is, the stage of the first occurrence of successful coordination *or* otherwise Stage 3. Toddler's received a score of 0 (*no success*) if they showed only uncoordinated behaviors (e.g., individual attempts to solve the task, performing role in absence of peer partner, exploring the box, leaving the box and exhibiting off-task activities, etc.) and did not succeed in activating the toy, that is, they were *unable* to activate the toy once. Children received a score of 1 (*low coordinated*) if they showed some coordinated actions (e.g., spontaneous coordinated pulls/pushes, pushing button or pulling the handle if peer partner is close by, appropriate posture and observation of partner's activities), but predominantly uncoordinated behavior. Children were awarded a score of 2 (*coordinated*) if they performed mostly coordinated activities and only a few episodes of uncoordinated actions. Finally, they were awarded a score of 3 (*very coordinated*) if they spontaneously performed their role correctly (latency <10 s) and showed no further uncoordinated activities. Interrater reliability for the level of coordination coding was good (Kendall's τ = 0.82). In addition, we used the coordination score as an indicator for toddlers' current performance experiences to analyze the role that these experiences in Phase 2 played in the toddler's subsequent motivation to collaborate (i.e., preference for joint activity) in Phase 3.

#### Preference for joint activity (Phase 3)

In Phase 3 (but also in Phase 2), we exclusively and exhaustively coded toddlers' observed activities by recording exact on- and offsets. Major categories were toddlers' individual and joint play during these phases (see also Table 1 for detailed descriptions of the coding scheme and the different categories). Moreover, toddlers could receive a “joint parallel play” activity coding during Phase 3 (but not in Phase 2); that is, toddlers played individually (e.g., each child played with an individual play box) but side by side, and they frequently communicated or shared attention with their peer partner. This specific (and somewhat unexpected) type of joint play was qualitatively different from individual play without referencing the partner. Interrater reliability for coding the toddlers' activity was moderate to good (Cohen's κ = 0.58). On the basis of the codings for toddlers' activities during Phase 3, we calculated the *preference for joint activity* score to specify toddlers' preference and persistence in performing joint activities compared to individual actions. To specify the degree of toddlers' commitment to joint activities, we calculated the ratio of the percentage of time spent playing jointly during Phase 3 (i.e., joint play = joint collaboration + joint parallel play) divided by the percentage of time being on task (= joint play + individual play + on task others). We used this *preference for joint activity* score for further analyses.

**Table 1:** **Coding scheme for toddlers' activities in Phases 2 and 3**.

**CATEGORIES AND DESCRIPTION**
***Joint collaboration***
Both children play jointly at a box (Phase 2: collaboration box; Phase 3: collaboration box or individual play boxes). The following criteria must be fulfilled:
• Both children sit at the same box and each child performs her or his role (i.e., both children are positioned correctly in front of the buttons/handles within arm's length).
• At least one child should push the button/pull the handle.
• Child A should *not* try to displace Child B in order to monopolize the apparatus and activate the train individually.
***Joint parallel play (Phase 3 only)***
Each child plays individually with one of the individual play boxes (e.g., Child A at Box 2 and Child B at Box 3). To receive the coding “joint parallel play,” children additionally need to communicate on the task and/or share attention (i.e., Child A watches Child B's activities at the neighboring box while playing herself/himself).
***Individual play***
Child plays individually at a box. The child shows no signs of waiting (else coding “on task others,” see below) or reengagement attempts. Examples:
• Child focuses on her or his box and pushes the button alone, while peer is off task.
• Child tries to activate the toy individually, for example, pushes both buttons/handles simultaneously, or unsuccessfully tries to activate the toy in Box 1 (collaboration box), pushes a peer away from a box to monopolize apparatus, etc.
***On task others (e.g., waiting, exploration, or other)***
Child waits on task at an apparatus (waiting), explores a box (e.g., sits on it, tries to peek behind it, climbs on the box, knocks on the Plexiglas, shakes the Plexiglas divider, etc.) or shows other on task activities not coded by the categories above.
***Off-Task***
Toddler's activities when distance to the boxes is greater than an out-stretched arm's length; that is, if child is approximately >.5 m away from a box, for example, child runs to the table and/or sits down close to mother, runs through the room, explores the room, etc.
***Not codable/visible***
Activities that could *not* be coded because children were out of range for the cameras.

#### Mothers' expectations

We asked the mothers to report their expectations regarding their children's cooperative social behaviors with a specially developed 19-item questionnaire. All items had to be rated on a 4-point scale ranging from 1 (*not at all*) to 4 (*total agreement*). The questionnaire was divided into the following four subscales: empathy/concern for others (6 items, e.g., “I expect my child to comfort other children if they are sad”), instrumental helping (5 items, e.g., “I expect my child to help when chores need to be done”), agreeableness (5 items, e.g. “I expect my child to get along well with others”), and sharing (3 items “I expect my child to share her/his belongings with others”). The internal consistencies of these subscales ranged from good to excellent (Cronbach's α between 0.79 and 0.89). Because mothers had a clear tendency to rate all items as important (overall *M* = 2.96, *SD* = 0.45), we centered the scores on the mean of all responses. For the final analyses, we used the mean-centered values for the subscales *empathy, instrumental helping, agreeableness*, and *sharing*.

#### Previous positive experiences

During the initial free play period (Phase 1), we coded the “quality of interactions” on a 5-point scale ranging from -2 (*hostile-aggressive*) to 2 (*positive-enjoying*) and the “number of interactions” on a 5-point scale ranging from 0 (*no interactions*) to 4 (*many interactions*) based on the toddlers' overall interaction. Interrater reliabilities were satisfactory (quality of interactions: Kendall's τ = 0.73; number of interactions: Kendall's τ = 0.65). Based on these two categories, we calculated a “*positive experiences*” score for further analyses by computing the product of the quality and the number of interactions (*positive experiences* = quality of interactions × number of interactions; range: -8 to 8). Thus, having many positive social interactions with the peer partner during free play resulted in a high positive experiences score, whereas having many negative interactions resulted in a low score. Note that the previous positive experiences score characterized the dyad; that is, both children within a dyad had the same positive experiences score.

#### Temperamental factors and mastery motivation

We assessed toddlers' temperamental dispositions (i.e., *shyness, sociability, fear, inhibitory control*) by applying a German version of Rothbart's Early Child Behavior Questionnaire (ECBQ, Putnam et al., 2006). Mothers had to rate the occurrences of the described behaviors for each item on a 5-point scale ranging from 1 (*never or rarely*) to 5 (*almost always or always*). The original questionnaire consists of 18 sub-scales and 200 items in total. Given our hypotheses, we focused on the relevant sub-scales regarding toddler's extraversion, negative affectivity and effortful control, namely: shyness (12 items, e.g. “… pull back and avoid the person?”), sociability (8 items, e.g. “… seek out the company of the child?”), fear (11 items, e.g. “… seem frightened by ‘monster’ characters?”), and inhibitory control (12 items, “… seem unable to wait for as long as 1 min?”). Internal consistencies were good to excellent (Cronbach's αs between 0.79 and 0.83). Moreover, we asked mothers to rate their toddler's motivation to master their environment or challenging tasks. Corresponding items were taken from the *mastery motivation* scale of the Infant–Toddler Social and Emotional Assessment (ITSEA, Carter et al., 2003; see Tietze et al., 2012, for the German version; example items: “Enjoys challenging activities,” “Keeps trying even when something is hard”, “Wants to do things for himself or herself”). The scale contains 5 items and describes occurrences that have to be rated on a 3-point scale ranging from 1 (*not or rather not*) to 3 (*often or very often never*). Its internal consistency was acceptable (Cronbach's α = 0.61).

## Results

### Preliminary analyses

We tested for potential differences in toddlers' collaboration between the two apparatuses. We found significant differences based on the apparatus (train box vs. disco-ball box) for level of coordination (*Mann*–*Whitney U* = 125, *p* < 0.01), but not for preference for joint activity [*t*_(43)_ = 1.90, *p* = 0.064]: Children's level of coordination was higher for the train box than the disco-ball apparatus. However, the apparatus type did *not* correlate significantly with any other variable (*r* between −0.26 and 0.14, *p* > 0.10, two-tailed). Based on these findings and for reasons of parsimony, we decided to drop apparatus type from further analysis models. In addition we checked, whether there were any significant differences in toddlers' collaboration between same-sex and mixed-sex dyads. Results indicated that this was not the case [coordination: *Mann-Whitney U* = 238.5, *p* = 0.75; joint activity preference: *t*_(43)_ = 0.08, *p* = 0.98]. Finally, none of the control variables (i.e., age, gender) correlated significantly with any of the collaboration variables (−0.07 < *r* < 0.22, *p* > 0.10, two-tailed). Hence, we dropped these variables from further analyses.

### Descriptives

**Figure 3:**
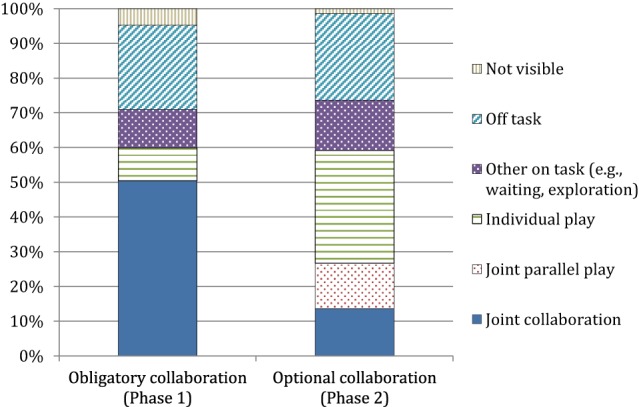
**Children's activities in Phase 2 and 3 as a percentage of total time**.

Figure 3 presents the average percentage of time children spent on the different activities during Phase 2 and Phase 3; that is, the amount of time on individual play, joint play (= joint parallel play + joint collaboration), on task others (i.e., waiting, exploration), or off task behavior. In Phase 2, children were engaged most of the time in “joint collaboration” (51.9%) and exhibited, on average, only short periods of individual play (9.7%). However, this pattern seemed to invert in Phase 3: Children spent approximately one-third of the time in individual play (32.5%) and only 13.6% in joint collaboration. Toddlers were relatively coordinated during the obligatory collaboration in Phase 2 (*M* = 1.63, *SD* = 0.95). Their average preference for joint activity value in Phase 3 with optional collaboration was 0.31 (*SD* = 0.29); that is, they spent around one-third of their on task time at the boxes playing jointly (i.e., in parallel or collaboratively). Parents reported medium levels for their toddlers' dispositional shyness (*M* = 2.39, *SD* = 0.67) and considerably high values for mastery motivation (*M* = 2.73, *SD* = 0.22). Concerning the average positive experiences values, we found that the dyads on average exhibited moderately positive-harmonious interactions during free play (*M* = 2.45, *SD* = 3.53).

### Correlations

To analyze the relations between toddlers' cooperative behavior (i.e., level of coordination, preference for joint activity) and the different factors introduced above, we first calculated zero-order correlations (see Table 2). As expected, shyness was associated negatively with both level of coordination and joint commitment. In addition, toddlers' dispositional fear was negatively related to preference for joint activity. Furthermore, mastery motivation correlated negatively, whereas initial positive experiences during free play correlated positively with preference for joint activity. Moreover, there were positive and mostly significant correlations between mothers' expectations regarding sharing and agreeableness, and negative correlations with mothers' expectations regarding empathy and both aspects of coordination. Finally, we found a significant positive association between level of coordination in Phase 2 and preference for joint activity in Phase 3 (*r*_Spearman_ = 0.67, *p* < 0.01). In addition, we calculated partial correlations between the collaboration variables (i.e., coordination, preference for joint activity) and the different factors (i.e., shyness, mastery motivation, initial positive experiences, mother's expectations): Partialling out apparatus type did not have any effect on the general significance pattern of correlations found above (see also Supplements). This further supported the initial decision to drop apparatus type from subsequent analyses.

**Table 2:** **Zero-order correlations between toddlers' cooperative behavior and variables on individual, dyadic, and social factors**.

	**Toddlers' cooperative behavior**	
	**Level of coordination (Phase 2)**	**Preference for joint activity (Phase 3)**
1.Shyness	−0.28^*^	−0.30^*^
2.Sociability	0.10	0.11
3.Fear	−0.24	−0.31^*^
4.Inhibitory control	−0.07	−0.14
5.Mastery motivation	−0.05	−0.35^**^
6.Previous positive experiences	0.09	0.43^**^
7.Parent's expect. sharing	0.32^*^	0.24
8.Parent's expect. agreeableness	0.27^*^	0.30^*^
9.Parent's expect. helping	−0.00	−0.06
10.Parent's expect. empathy	−0.44^**^	−0.41^**^

*Coordination: r_Spearman_; Preference for joint activity: r_Pearson_. Because of dyadic nonindependence in the data, we alternatively calculated correlations with a more robust bootstrap procedure yielding similar findings*.

* *p < 0.05, one-tailed*.

** *p < 0.01, one-tailed*.

### ICC and modeling dyadic data

On the basis of the significant zero-order correlations, we computed separate models for (a) level of coordination (Phase 2) and (b) preference for joint activity (Phase 3). In advance, we checked whether data were dependent within dyads so that nonindependence could be taken into account in the model building process. Nonindependence is frequently present in collaborative situations because children influence each other in their behaviors, for example, by means of communication or by observing their partners' actions (e.g., Hunnius et al., 2009; Hamann et al., 2012). Moreover, Kenny et al. (2006) generally recommend assuming nonindependence by default for sample sizes around *N* = 25 dyads.

The ICC coefficients typically used for testing nonindependence revealed dyadic dependences in the present data on coordination and preference for joint activity (ICC = 0.80 for level of coordination and 0.51 for preference for joint activity; Kenny et al., 2006). If we were to simply ignore these dependencies, we would run the risk of alpha inflation. Consequently, we did not use standard models (e.g., ordinary linear regression), and applied more conservative modeling techniques to estimate significance. This approach should minimize Type I errors and increase confidence in the significance of the findings. Given the nested structure and dyadic dependence of the data (Kenny et al., 2006), linear mixed models are recommended with corresponding modeling techniques (i.e., GLMM, LMM). Therefore, we used generalized linear mixed models (GLMM; e.g., Heck et al., 2012; Loeys et al., 2014) for level of coordination, because these data were ordinally scaled, and linear mixed models (LMM; e.g., Kenny et al., 2006; Kenny and Kashy, 2011) for preference for joint activity, because these were interval-scaled data. Dyad was always set as a random effect in each of the models to control for the nonindependence of the data. Furthermore, we chose the fixed effect variables (independent variables) based on the significant zero-order correlations with the DVs (see Table 2 above). For the model building process, we generally started with a full model and then reduced the model in successive steps. The aim of this modeling process was to find a parsimonious model that would explain the data (i.e., the variance in toddlers' coordination and preference for joint activity) equally well or better.

### Level of coordination (Phase 1)

The starting model for coordination (GLMM) was a full model with shyness and parent's expectations regarding agreeableness, empathy, and sharing as predictors. This model was marginally significant (*F*_4, 39_ = 2.54, *p* = 0.055, model fit: *AICc* = 737.16; see also Table 3). In the next step, we removed empathy from the model in order to improve its parsimony, because this predictor was well above any meaningful significance level (*p* > 0.20) and thus did not seem to make any substantial contribution to explaining the variance in toddlers' coordination. The reduced model had a significantly improved fit (*AICc* = 733.70; likelihood-ratio-test [LR test]: X^2^(1) = 3.969, *p* < 0.05) and also attained significance (*F*_3, 40_ = 2.99, *p* < 0.05). In particular, maternal expectations regarding sharing significantly predicted toddlers' level of coordination (see Table 4), and, furthermore, children's shyness became a marginally significant predictor. Thus, children whose parents had higher expectations regarding their sharing had better coordination whereas children with higher dispositional shyness had lower levels of coordination.

**Table 3:** **Full model for toddlers' level of coordination in Phase 2 with parameter information for fixed effects of the general linear mixed model**.

**Predictors**	***b***	***SE***	**Sig**.
Shyness	−0.92	0.48	0.07
Mothers' expectations agreeabl.	1.72	1.04	0.11
Mothers' expectations sharing	1.79	0.96	0.07
Mothers' expectations empathy	0.37	2.16	0.86

*n = 23 dyads; b, unstandardized regression coefficient; SE, standard error*.

**Table 4:** **Final parsimonious model for toddlers' level of coordination in Phase 2 with parameter information for fixed effects of the general linear mixed model**.

**Predictors**	***b***	***SE***	**Sig**.
Shyness	−0.94	0.49	0.059
Mothers' expectations agreeabl.	1.65	1.08	0.135
Mothers' expectations sharing	1.64	0.80	0.045

*n = 23 dyads; b, unstandardized regression coefficient; SE, standard error*.

### Preference for joint activity (Phase 2)

We analyzed the interval data on toddlers' preference for joint activity with linear mixed models. Again, predictor selection was based on significant zero-order correlations (see Table 2): The full model included previous positive experiences, shyness, fear, mastery motivation, and parent's expectations regarding agreeableness and empathy as fixed effects. Moreover, we added level of coordination from Phase 2 as an additional fixed effect to investigate the role of previous performance (i.e., experiences of competence and success) in toddlers' subsequent willingness to cooperate (i.e., preference for joint activity) in Phase 3. The full model revealed coordination and previous positive experiences as significant positive predictors (see Table 5). In addition, mastery motivation was a significant negative predictor; that is, children with higher levels of mastery motivation showed less commitment to joint activites (i.e., preferred individual play to joint play). Based on the findings in the full model, we decided to drop shyness, fear and mothers' expectations because they clearly exceeded meaningful significance levels (all α > 0.20). The reduced model indicated that toddlers' mutual sympathy during free play and their level of coordination in Phase 2 promoted their commitment to joint activites in Phase 3, whereas children's mastery motivation seemed to conflict with their willingness to collaborate (see Table 6). A model comparison indicated that the reduced model showed a significant improvement in explaining toddlers' preference for joint activity [model comparison: LR test: *X*^2^_(4)_ = 13.72; *p* < 0.01]. Hence, the findings in the reduced model were comparable to the full model. However, model reduction led to more parsimony and further supported the assumption that the excluded predictors made no substantial contribution to explaining the variance in toddlers' preference for joint activity.

**Table 5:** **Full model with parameter information for fixed effects in the linear mixed model on toddlers' preference for joint activity in Phase 3**.

**Predictors**	***b***	***SE***	**Sig**.
Shyness	−0.02	0.04	0.54
Fear	−0.02	0.05	0.79
Mastery motivation	−0.23	0.10	0.04
Coordination (Phase 2)	0.15	0.04	0.00
Previous positive experiences	0.03	0.01	0.01
Mothers' expectations agreeabl.	−0.04	0.09	0.69
Mothers' expectations empathy	−0.12	0.10	0.25

*n = 23 dyads; b, unstandardized regression coefficient; SE, standard error*.

**Table 6:** **Final parsimonious model with parameter information for fixed effects in the linear mixed model on preference for joint activity in Phase 3**.

**Predictors**	***b***	***SE***	**Sig**.
Coordination (Phase 2)	0.16	0.04	0.00
Previous positive experiences	0.03	0.01	0.01
Mastery motivation	−0.21	0.09	0.03

*n = 23 dyads; b, unstandardized regression coefficient; SE, standard error*.

## Discussion

The major goal of this study was to identify the relative contribution of individual, dyadic, and social factors in explaining the interindividual differences in toddlers' level of coordination and preference to act jointly indicating their commitment to joint activities. We found that each of these different factors was associated with children's collaborative efforts. These findings are unique in the sense that they reveal the complexity of explaining individual differences in human collaboration and extend former approaches by simultaneously looking at various contributing factors to toddlers' collaborative efforts on multiple conceptual levels.

### Social factors: Socialization & toddlers' collaboration

One of the main finding was that mothers' expectations regarding their children's social behaviors were related to the displayed collaboration with an unfamiliar peer. In particular, mothers' expectations regarding their toddler's sharing were associated positively with children's coordination; that is, the competence to work jointly toward a shared goal. Hence, these findings indicate that parents' socialization goals may promote 3-year-olds' collaborative efforts. Moreover, these goals seem to build on a biologically prepared human disposition to collaborate (e.g., Warneken and Melis, 2012). But why was it particularly the expectations regarding sharing that predicted toddlers' collaborative performance in the corresponding model? A couple of recent findings reveal that sharing and cooperation are two closely interrelated domains. For example, Hamann et al. (2011); Hamann et al. (2014) have found that collaboration actually elicits equal sharing in young children. Moreover, Hay (1979) found that 2-year-olds spontaneous sharing with mothers was associated with their collaborative behavior in social interchanges between child and parent. According to Tomasello (2009), this interrelation might have evolved as a specifically human heritage, because our Pleistocene hunter and gatherer ancestors acted in line with the motto: “First hunt the spoil together, and then split it!” In contrast, chimpanzees tend to avoid collaboration because they anticipate a subsequent sharing conflict and/or a loss of spoil based on the partner's dominance level. Thus, corresponding socialization experiences concerning sharing might have an effect on collaboration via a phylogenetically evolved relation between mutualistic collaboration and altruistic sharing (e.g., Tomasello et al., 2012). Findings from Callaghan et al. (2011) point to the idea that there might be various (culture-specific) relations between parent's socialization goals on cooperation and toddlers' collaborative activities: For example, Indian mothers specifically stated the relevance of collaboration for social approval and fostered the concern for others' needs in their children. Peruvian mothers stressed the benefits for building character and helping community. In brief, social factors (i.e., parent's expectations) compose one elementary element in the ontogeny of young children's collaboration. However, a future perspective might be to elucidate which socialization practices explicitly mediate parenting goals regarding their children's cooperative behaviors in different cultures.

A potential methodological limitation in interpreting the results on mother's expectations toward toddler's cooperative behaviors and the role of socialization in the development of children's collaboration might be the objection that mothers simply indicated their observations of toddler's actual cooperative behavior at home or during everyday encounters. On the basis of such an interpretation a positive association between mothers' ratings and toddlers' collaborative behavior in the lab seems to be a trivial finding. However, in the scope of a larger project we also assessed mothers' ratings of toddlers' actual cooperative behaviors at home for this study's sample. In a *post-hoc* analysis we calculated the correlations between mothers' expectations and mothers' rating of the actual cooperative behaviors and found no to moderate associations between these two different measures (−0.09 < *r* < 0.35). That is, these ratings were by no means tautological (but rather idiosyncratic) and mothers seemed to be very sophisticated in differentiating between their personal expectations regarding their children's behaviors and the actually displayed behaviors.

### Dyadic factors: The role of shared previous experiences

Major results regarding the role of dyadic factors were that unfamiliar peer dyads with positive experiences during initial free play showed a higher preference for joint activity later on (e.g., preference to collaborate instead of playing individually with an apparatus) even when controlling for dispositional shyness. In addition, the more coordinated toddlers were during obligatory collaboration, the more persistent they were in acting jointly during optional collaboration. This finding could be interpreted in the sense that toddlers' previous performance and associated experiences of competence in jointly solving a problem, might have further promoted their motivation to collaborate in a subsequent optional collaboration situation. However, an alternative (more simplistic) interpretation might be that this association can be explained on the basis of a common underlying factor: Specifically skilled and motivated toddlers (e.g., socially more competent children) might put more effort in both situations leading to higher levels of coordination as well as persistence in joint activities. This interpretation cannot be ruled out on the basis of the present study. However, it seems plausible that both processes might actually add up in a mutually reinforcing manner and thus both account for the individual differences in toddlers' preference for joint activities. In addition, it is important to point out here that toddler's coordination and preference for joint activities obviously differ on a conceptual level: Whereas the former denotes toddlers' skills to coordinate own behaviors with others in an efficient fashion (i.e., showing mutually responsive goal-oriented behaviors), the latter is indicative for toddlers' willingness to play jointly at the boxes and to prefer joint activity over individual goal attainment. Moreover, the corresponding situations (i.e., Phase 2 and 3) are distinct in the following respect: Whereas collaboration was obligatory in Phase 2 (e.g., toddlers were increasingly instructed to collaborate by E1) children's collaboration in Phase 3 was optional, that is, toddlers could choose between individual or joint play and interacted freely during this period. On the background of these differences, associations between toddlers' coordination and preference for joint activity appear to be meaningful (and not trivial). In a similar vein, the relations between toddlers' initial positive experiences and later preference for joint activity could be discussed critically as well: For example, socially more competent children could have more positive interactions during free play and might also prefer to act jointly during the optional collaboration phase. However, the modeling results yielded that previous positive experiences and toddlers' coordination both contributed individually to explaining the interindividual differences in toddlers' preference for joint activities. This finding seems counterintuitive to an explanation of the results structure based on a common underlying factor. To sum up, despite the discussed potential methodological limitations the results clearly indicate that establishing a commitment to a joint activity during optional collaboration is positively related to previous coordination efforts and to spontaneous positive social interactions among unfamiliar interaction partners.

Finally, the emerging question on the basis of these results is: Why might previous experiences with an unfamiliar interaction partner be associated with a preference to collaborate with this partner instead of playing individually? One, admittedly speculative, interpretation is that the positive interactions during free play likely foster a positive and trustful relationship between the interaction partners in the sense: “You are a nice and reliable interaction partner.” This claim is particularly interesting against the background of some recent ideas on proximate and ultimate processes underlying human cooperation (e.g., Tomasello et al., 2012; Baumard et al., 2013): In accordance with various evolutionary approaches these scholars postulate that for an evolutionarily stable development of cooperation, it is important that actors protect themself from being exploited by free riders, particularly in encounters with strangers. Beyond the level of partner control mechanisms (e.g., punishing noncollaborators, tit-for-tat strategy), the detection and choice of reliable and trustworthy interaction partners might be an elementary mechanism for this evolution of cooperation. So far we can only speculate about the proximate cognitive and/or affective processes underlying the detection of reliable interaction partners. In the literature, different homophily tags are currently discussed as potential markers for kinship or group membership, for example, behavioral similarity, accent, or facial self-similarity (e.g., Kurzban et al., 2001; Krupp et al., 2011; Dunfield et al., 2013; Haun and Over, 2014). Future research should address these issues more directly by performing systematic analyses of the relative importance of these different markers for the ontogenetic (and phylogenetic) development of human cooperation. One promising idea is to create experimental settings in which children can actively choose among different unfamiliar cooperation partners or in which the level of liking is manipulated experimentally. Furthermore, it would be interesting to see how these different preferences in collaborative tasks might spill over to behaviors in other cooperative situations, that is, sharing, helping, or comforting (see also Dunfield et al., 2013; Gräfenhain et al., 2013).

### Individual factors: Mastery as a conflicting motive

We found that children's mastery motivation was negatively associated with toddlers' preference for joint activity. Mastery motivation might be indicative for an emerging achievement motivation or personality factor (Denham et al., 2009). Thus, our findings show that specific individual factors, namely the motivation to master the environment and to solve challenging tasks autonomously, might interfere with a child's motivation to collaborate. The findings in the models furthermore highlight that by no means all children build a similar degree of motivation to act jointly, but that there is a significant amount of interindividual variation in preference for joint activity. For example, some children might actually be interested in mastering a task on their own. In order to attain this individual goal, they potentially use others as a social tool. This interpretation gathers some support from the finding that joint collaboration in toddlers dropped from Phase 2 to Phase 3 [postanalysis: *t*_(45)_ = 7.28, *p* < 0.001; two-tailed], that is, some children might have lost their interest in collaborating with a partner as soon as they had the option to operate a similar box individually. On an anecdotal level, we observed that apologies for stopping collaboration (i.e., hesitance or verbal excuses when leaving partner) hardly occurred during Phase 3. This would indicate that these children had not established a commitment to do things together during Phase 2, but had simply used the other partner as a social tool to attain their own individual goals (see, Gräfenhain et al., 2009). Alternatively, this drop could indicate an increase in curiosity regarding the new boxes (although the new boxes were almost identical to the first box in Phase 2 and toddlers already had the opportunity to explore this box before) or a lack of scaffolding by E1 during Phase 3. Finally, one could also speculate to what degree toddlers' mastery motivation is influenced by sociocultural factors specifically promoted in autonomy-oriented western industrialized cultural contexts. For example, the provision of toys—characteristic for western-industrialized contexts (see Keller, 2007)—is know to be positively associated with toddlers' development of mastery motivation in these contexts (Busch-Rossnagel, 1997). Moreover, mothers' parenting behaviors supporting their offspring's autonomy seem to foster toddlers' mastery motivation in these contexts as well (e.g., Kelley et al., 2000). In this regard it might be fruitful to differentiate between social and object-oriented mastery motivation and investigate the relation between toddlers' mastery motivation and collaborative activities in different cultures. Future research could analyze the relation between parents' socialization practices and goals, toddlers' mastery motivation, and toddlers' willingness to collaborate more thoroughly in different sociocultural contexts.

## Conclusion

In summary, this study highlights the considerable interindividual variation in toddlers' cooperation with unfamiliar peers. This can be attributed to various variables at multiple conceptual levels, namely social, dyadic, and individual factors. Previously shared experiences within a dyad of unfamiliar partners, for example, initial positive interactions and efforts in coordination, seem to promote toddlers' commitment to joint activities during optional collaboration, whereas socialization experiences might affect toddlers' level of coordination. A future challenge for developmental research is to find out more about the onset, ontogeny, and interplay of these manifold factors that build on the human's natural predispositions to cooperate and that potentially contribute to the emergence of cooperation.

### Conflict of interest statement

The authors declare that the research was conducted in the absence of any commercial or financial relationships that could be construed as a potential conflict of interest.

## Footnotes

^1^In the following, we shall stick to the term collaboration (or joint collaborative activities). But, we shall also use the term cooperation interchangeably, thereby referring mostly to mutualistic cooperation.

^2^Please note that the definition of children's mastery motivation provided here suits best to its early forms, that is, between 6 months and 6 years of age (see also Morgan et al., 2009), and thus might differ from conceptions of mastery motivation or achievement later in ontogeny (e.g., during late childhood or adolescence).
