# Supplementary material for: Explaining interindividual differences in toddlers' collaboration with unfamiliar peers: individual, dyadic, and social factors
Source: Front Psychol. 2015 May 1;6:493. doi: 10.3389/fpsyg.2015.00493 (PMC4416470; doi:10.3389/fpsyg.2015.00493)
Supplement: Supplementary file 1 [file Table1.DOCX]

Supplements A

Results: Partial Correlations

Table X.1

*Zero-order and partial correlations (controlling for apparatus type) between toddler’s cooperative behavior and variables on individual, dyadic, & social factors*

|  | Toddler’s cooperative behavior | |
| --- | --- | --- |
|  | Level of coordination  (Phase 2) | Preference for joint activity  (Phase 3) |
| 1. Shyness | -.28*/-.32* | -.30*/-.31* |
| 1. Sociability | .10/.05 | .11/.08 |
| 1. Fear | -.24/-.25* | -.31*/-.31* |
| 1. Inhibitory control | -.07.03 | -.14/-.09 |
| 1. Mastery motivation | -.05/-.09 | -.35**/-.35* |
| 1. Previous positive experiences | .09/.26* | .43**/.46** |
| 1. Mothers’ expect. sharing | .32*/.24^+^ | .24/.18 |
| 1. Mothers’ expect. agreeableness | .27*/.25^+^ | .30*/.25^+^ |
| 1. Mothers’ expect. helping | -.00/.00 | -.06/-.01 |
| 1. Mothers’ expect. empathy | -.44**/-.32* | -.41/-.38** |

*Note.* Coordination: *r_Spearman_*; Preference for joint activity: *r_Pearson_*. Values behind slash denote the partial correlations. Because of the dyadic non-independence in the data, we alternatively calculated correlations with a more robust bootstrap procedure yielding similar findings.

^+^ *p* < .10, 1-tailed. * *p* < .05, 1-tailed. ** *p* < .01, 1-tailed

Supplements B

Results: Intercorrelations

Table X.2

*Zero-order correlations among children’s level of coordination, preference for joint activity, & other variables*

|  | 2 | 3 | 4 | 5 | 6 | 7 | 8 | 9 | 10 | 11 | 12 |
| --- | --- | --- | --- | --- | --- | --- | --- | --- | --- | --- | --- |
| 1. Coordination | **.671^**^** | **-.473^**^** | **-.275^*^** | -.046 | .091 | **.321^*^** | **.265^*^** | -.004 | **-.437^**^** | -.073 | .061 |
| 1. Preference for joint activity |  | **-.278^*^** | **-.299^*^** | **-.353^**^** | **.432^**^** | .240 | **.296^*^** | -.058 | **-.408^**^** | .081 | .217 |
| 1. Apparatus |  |  | -.013 | .054 | .005 | -.235 | **-.257^*^** | .219 | .190 | .140 | .142 |
| 1. Shyness |  |  |  | .063 | -.179 | .149 | -.237 | -.075 | -.100 | -.201 | -.123 |
| 1. Mastery motivation |  |  |  |  | .037 | .159 | -.016 | -.051 | .139 | **-.302^*^** | -.149 |
| 1. Previous positive experience |  |  |  |  |  | -.006 | .215 | -.142 | -.077 | .071 | -.091 |
| 1. Mothers’ expect. sharing |  |  |  |  |  |  | .116 | -.183 | **-.632^**^** | -.077 | **-.271^*^** |
| 1. Mothers’ expect. agreeabl. |  |  |  |  |  |  |  | **-.507^**^** | -.234 | .129 | -.024 |
| 1. Mothers’ expect. helping |  |  |  |  |  |  |  |  | .137 | -.133 | **.275^*^** |
| 1. Mothers’ expect. empathy |  |  |  |  |  |  |  |  |  | .011 | .011 |
| 1. Age |  |  |  |  |  |  |  |  |  |  | .102 |
| 1. Gender |  |  |  |  |  |  |  |  |  |  |  |

*Note.* To receive more robust results (because of the potential non-independence of dyadic data) we alternatively calculated correlations with a bootstrap procedure yielding similar findings. Coordination: *r*_Spearman_; Other variables: *r*_Pearson_

* *p* < .05, 1-tailed. ** *p* < .01, 1-tailed
